# Vitamin D protects glomerular mesangial cells from high glucose-induced injury by repressing JAK/STAT signaling

**DOI:** 10.1007/s11255-020-02728-z

**Published:** 2021-05-03

**Authors:** Yiya Yang, Yuting Lei, Yumei Liang, Shuangshuang Fu, Congjun Yang, Kanghan Liu, Yinyin Chen

**Affiliations:** Department of Nephrology and Laboratory of Kidney Disease, Changsha Clinical Research Center for Kidney DiseaseHunan Clinical Research Center for Chronic Kidney Disease, Hunan Provincial People’s Hospital, Hunan Normal University, 61#, Jiefang West Road, Changsha, 410005 Hunan Province People’s Republic of China

**Keywords:** Reactive oxygen species, High glucose, Vitamin D, JAK/STAT, Glumerular cell

## Abstract

**Aim:**

High glucose (HG) induces the production of transforming growth factor (TGF)-β and reactive oxygen species, which further activates JAK/STAT signaling and promotes the synthesis of matrix proteins, contributes to the pathophysiological processes of diabetic nephropathy. This study aims to investigate the protection role of vitamin D (VD) in the kidney in high glucose condition.

**Methods:**

Rat glomerular mesangial cells were cultured in high glucose medium, with or without VD or VD receptor (VDR) siRNAs treatment. The levels of TGF-β and fibronectin were detected by qRT-PCR, immunoblotting and enzyme-linked immunosorbent assay (ELISA). The levels of phosphorylated JAK2, STAT1 and STAT3, and JAK/STAT signaling downstream genes were examined by immunoblotting and qRT-PCR.

**Results:**

In rat glomerular mesangial cells, VD treatment can repress the tyrosine phosphorylation of JAK2, STAT1 and STAT3. VD inhibited TGF-β and fibronectin expression which was rescued by vitamin d receptor (VDR) siRNA and STATs inhibitor perficitinib. The JAK/STAT signaling downstream protein coding genes including SOCS1, SOCS3 and type IV collagen were repressed by VD. Meanwhile, the expression of non-coding RNAs such as miR-181a, miR-181b, was repressed by VD, and the expression of miR-34a and Let-7b was upregulated by VD.

**Conclusion:**

Vitamin D (VD) treatment inhibits the function of HG on fibronectin production through regulating JAK/STAT pathway. These results provide direct evidences that VD protects glomerular mesangial cells from high glucose-induced injury through repressing JAK/STAT signaling, which has the potential for clinical DN treatment.

**Supplementary Information:**

The online version contains supplementary material available at 10.1007/s11255-020-02728-z.

## Introduction

Diabetic nephropathy (DN) is a common complication usually found in patients with diabetes mellitus (DM), which is accounting for chronic renal failure [1]. Although it is reported that abnormal renal hemodynamics, hyperglycemia-induced metabolic disorders, and the imbalance of vasoactive substances contribute to the development of DN, the underlying mechanisms for diabetic nephropathy are still not totally unveiled [2, 3]. Evidences indicate that the production of reactive oxygen species (ROS) is increased during hyperglycemia and the oxidative stress play a significant role during the development and progression of DN [4, 5]. ROS can stimulate the activity of the known STAT kinases JAK2 and TYK2, and the activation of the JAK/STAT signaling cascade further stimulates excessive proliferation and growth of glomerular mesangial cells, contributing to diabetic nephropathy [6, 7].

Numerous evidences indicate that hyperglycemia can also induce an increase of transforming growth factor-β1 (TGF-β) expression in cultured mesangial cells and diabetic mouse models [8–10]. Increased renal production of TGF-β was also observed in patients with diabetes and DN [11, 12]. TGF-β1 is a multifunctional cytokine the overexpression of which will enhance the synthesis and cross-linking of extracellular matrix (ECM) [13, 14]. Researchers found that neutralization of TGF-β prevented glomerulosclerosis and interstitial fibrosis, and reduced the expression of many ECM genes including fibronectin and type IV collagen in diabetic mice [15, 16].

Fibronectins are a group of large glycoproteins which are encoded by a single gene FN1. Fibronectins are key components of extracellular matrix which promote cell–cell interactions. A significant increase of fibronectin was reported in most types of glomerulopathy including DN, which contributes to aberrant thickening of glomerular basement membranes and excessively amassing of mesangial matrices [17–19].

Vitamin D has calciotropic and pleiotropic effects mediated through vitamin D receptor (VDR) expressed in different organs including kidney [20]. The VD deficiency is associated with increased urinary albumin excretion, as well as an increase in the prevalence of cardiovascular disease and mortality in patients with chronic kidney disease in the general population [21, 22]. Vitamin D was reported can inhibit JAK/STAT signaling in hepatocytes and T cells [23, 24]. Growing evidence indicates that VD deficiency could be a contributing factor in the development of both type 1 and type 2 diabetes. Evidences indicate that VD treatment improves glucose tolerance and insulin resistance by regulating insulin secretion [25–27]. However, whether VD protects kidney from HG-induced ROS injury, and what is the underlying mechanism are unclear. In the present study, we confirmed and examined the function of VD on the JAK/STAT signaling, TGF-β production and the expression of fibronectin in glomerular mesangial cells.

## Materials and methods

### Cell culture

Glomerular mesangial cells were isolated from rat glomeruli as previously described [28]. Briefly, glomeruli were obtained from male Sprague–Dawley rats and incubated with 250 units/mL collagenase I (SCR103, Sigma–Aldrich, St. Louis, MO, USA) for 30 min at 37 ℃. Glomerular mesangial cells were plated on tissue culture dishes in DMEM (11966025, Gibco, Thermo Fisher Scientific, Inc., Waltham, MA, USA) either with normal concentration glucose (NG) (A2494001, Thermo Fisher Scientific, Inc., Waltham, MA, USA) (5.5 mmol/L) or with high concentration glucose(HG) (25 mmol/L) supplemented with 10% fetal bovine serum (26140, lot 1566368, Gibco, Thermo Fisher Scientific, Inc, Waltham, MA, USA), 100 IU/ml penicillin and 10 mg/mL streptomycin (15140122, Gibco, Thermo Fisher Scientific, Inc., Waltham, MA, USA). All cells were maintained at 37 ℃ under an atmosphere of 5% CO2.

### ROS detection

- (a) The total intracellular superoxide and hydroxyl radical in cells was detected using Cellular Reactive Oxygen Species Detection Assay Kit (ab186027, Abcam, Cambridge, UK), following the manufacturer’s instructions. Briefly, cells were seeded into 96-well plate with 2 × 10^4^ cells per well. Add 100 µL/well of ROS Red Working Solution into the plate. Incubate cell plate in a 37 ℃/5% CO_2_ incubator for 1 h, and then detect the fluorescence signal by microplate reader.
- (b) Intracellular hydrogen peroxide detection. Cells were plated into 12-well plates with different treatments. Each well was loaded with 30 μm of the H2O2-sensitive fluorescent probe 2′,7′-dichlorofluorescein diacetate (DCF-DA; Molecular Probes, Leiden, The Netherlands) and then the plate was plated at 37 ℃ for 4 h. The cells were washed with PBS for three times and then the intracellular fluorescence was quantified using flow cytometry.
- (c) The level of superoxide anion was determined by ethidium fluorescence. Briefly, cells were seeded into 12-well plates wit different treatments. The cells were incubated with 1 mM hydroethidine at 37 ℃ for 5 min and then washed three times by PBS. The cells were then resuspended and subjected to flow cytometry analysis.

### Cell transfection

Small interfering RNA (siRNA) targeting VDR was transfected into cells using Lipofectamine 2000 Transfection Reagent (Thermo Fisher Scientific, Inc., Waltham, MA, USA), following the manufacturer’s instructions. siVDR sequence: 5′- rCrCrArArGrCrUrArUrCrUrGrArArGrArArCrArArCrArGCA-3′ and 5′- rUrGrCrUrGrUrUrGrUrUrCrUrUrCrArGrArUrArGrCrUrUrGrGrGrC-3'.

### Enzyme-linked immunosorbent assay (ELISA)

The level of secreted TGF-β and fibronectin proteins under the different experimental conditions were quantified using commercial ELISA kits following the manufacturer’s instructions. Rat TGF beta 1 ELISA Kit (ab119558, Abcam, Cambridge, UK) (sensitivity of the kit: higher than 7.8 pg/mL). Rat Fibronectin ELISA Kit (ab108850, Abcam, Cambridge, UK) (sensitivity of the kit: higher than 75 pg/mL).

### Immunoblotting

Protein samples were quantified using a Pierce BCA Protein Assay Kit (Thermo Fisher Scientific, Inc., Waltham, MA, USA), according to the manufacturer’s protocols. Protein samples were heated with SDS sample buffer (0.25 M Tris–HCl, pH 6.8, 0.5 M DTT, 10% SDS, 50% glycerol and 0.5% bromophenol blue) at 95 °C for 5 min, a total of 30 μg were loaded per lane and then separated on 15% SDS-PAGE and electroblotted onto polyvinylidene fluoride membranes (GE10600023, GE Healthcare Life Sciences, Logan, UT, USA). Subsequently, the membranes were incubated with one of the primary rabbit polyclonal antibodies at a dilution of 1:1,000 (Abcam) or anti-β-actin rabbit monoclonal antibody at a dilution of 1:5,000 (Abcam) overnight at 4 ℃. Immunoreactive bands were detected by incubation with horseradish peroxidase-conjugated goat anti-rabbit (Abcam) at a dilution of 1:5,000 for 2 h at room temperature. Detection by chemiluminescence was performed using a Pierce enhanced chemiluminescence kit (Thermo Fisher Scientific, Inc., Waltham, MA, USA).

#### Antibody information

Rabbit anti-fibronectin polyclonal antibody (ab2413, Lot. No. GR3261228-3, Abcam, Cambridge, UK); rabbit anti-phospho-JAK2 monoclonal antibody (ab32101, Lot. No. GR321849-8, Abcam, Cambridge, UK); rabbit anti-JAK2 monoclonal antibody(ab108596, Lot. No. GR261730-3, Abcam, Cambridge, UK); rabbit anti-VDR monoclonal antibody (ab109234, Lot. No. GR223547-2, Abcam, Cambridge, UK); rabbit anti-STAT1 monoclonal antibody(ab234400, Lot. No. GR323412-8, Abcam, Cambridge, UK); rabbit monoclonal anti-STAT1 (phosphor-Y701) antibody (ab109457, Lot. No. GR311243-6, Abcam, Cambridge, UK); rabbit anti-STAT3 monoclonal antibody (ab68153, Lot No. 32103-2, Abcam, Cambridge, UK), rabbit anti-STAT3 (phosphor-Y705) monoclonal antibody (ab76315, Lot. No. 312232-2, Abcam, Cambridge, UK), rabbit anti-β-actin polyclonal antibody (ab8227, Lot. No. 213113-8, Abcam, Cambridge, UK).

### RNA extraction and RT-qPCR

Total RNA from cells was extracted using a miRNeasy Mini kit (217004, Qiagen, Hilden, Germany) following the manufacturer’s instructions. Only samples with the appropriate absorbance measurements (A260/A280 of ~ 2.0, and A260/A230 of 1.9–2.2) were considered for inclusion in the present study. RT-qPCR was applied to detect the relative expression levels of selected mRNAs and miRNAs. The reaction conditions were as follows: 95 ℃ for 10 min, followed by 40 cycles of 95 ℃ for 15 s and 60 ℃ for 1 min. To compare the miRNA levels between different samples, the 2-ΔΔCq method was used. The level of GAPDH was used as loading control for mRNAs and U6 snRNA was used for miRNAs normalization. A total of three triplicate experiments were performed in each case.

### Statistical analysis

Statistical analysis was performed using SPSS software version 19.0 (IBM Corp., Armonk, NY, USA) and data are presented as the mean ± standard. The data were analyzed by Shapiro–Wilk test to examine whether the data are normal distributed. Results were then analyzed using one-way ANOVA Tukey's honestly significant difference (HSD) post hoc test. *P* < 0.05 was considered to indicate a statistically significant difference.

## Results

**Fig. 1:**
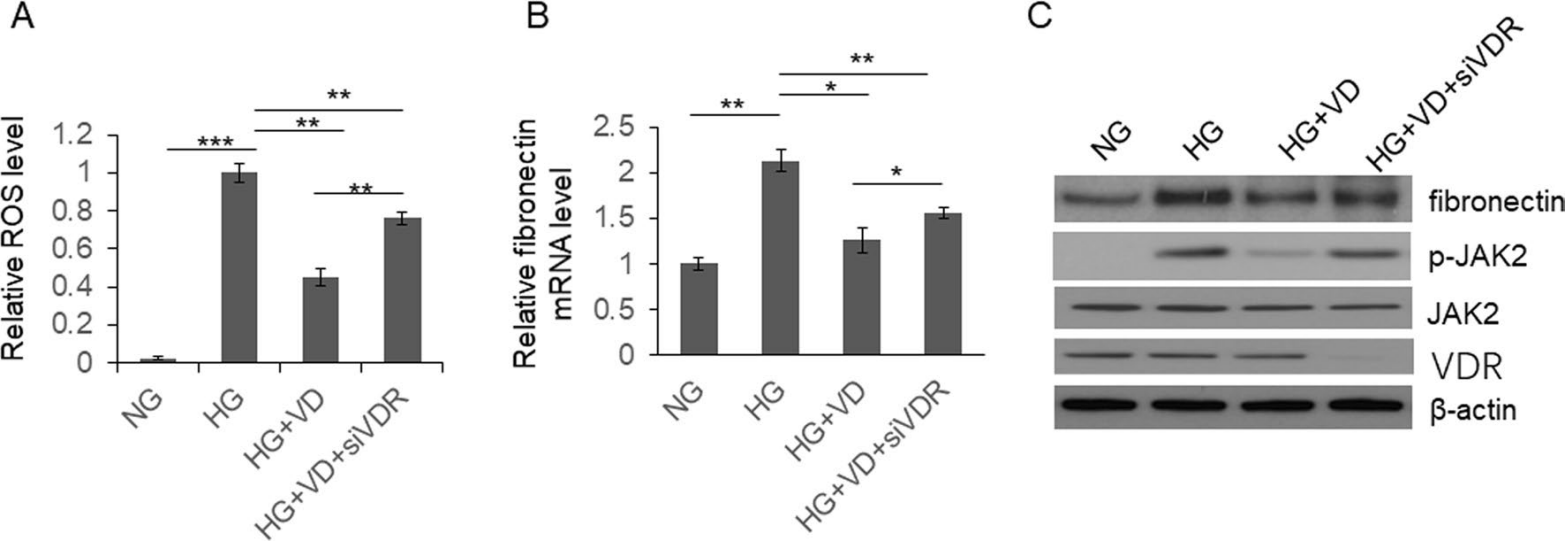
VD repressed ROS and fibronectin production induced by HG. Rat glomerular mesangial cells were cultured under different conditions with or without siVDR transfection for 48 h, and then subjected to intracellular ROS detection (**a**), or subjected to qRT-PCR (**b**) or immunoblotting (**c**) to examine the expression of fibronectin. Results were analyzed by one-way ANNOVA and *p* < 0.05 was considered significant. ***p < 0.001, **p < 0.0, *p < 0.05

To investigate the mechanisms of diabetic nephropathy, we compared the total ROS level in glomerular mesangial cells treated with normal and high glucose. As shown in Fig. 1a, high glucose significantly alleviated the ROS level in glomerular mesangial cells, which was partially reduced by vitamin D. Meanwhile, when VDR was knocked down by siRNA, the effect of VD on ROS repression was partially inhibited. These results indicated that VD/VDR signaling play protective roles in glomerular mesangial cells in high glucose environment. Subsequently, the intracellular hydrogen peroxide and superoxide anion were detected by flow cytometry separately to investigate which ROS subtype is VD/VDR signaling is related. As shown in Fig.S1, vitamin D treatment reduced the level of both hydrogen peroxide and superoxide in glomerular mesangial cells. When VDR was knocked down, the protective role of vitamin D is reduced, indicating that VD/VDR signaling protected glomerular mesangial cells from the injury of high glucose-induced hydrogen peroxide and superoxide.

Accumulation of extracellular matrix (ECM) proteins, including fibronectin, is one of the main causes of glomerulosclerosis in diabetic nephropathy. To understand the roles of HG on ECM accumulation, the mRNA level expression of fibronectin in were examined by qRT-PCR. As shown in Fig. 1b, the fibronectin expression in mesangial cells was significantly increased by HG. When the cells were treated by VD, the effect of HG on fibronectin elevation was almost totally repressed. Meanwhile, in the VDR knockdown cells, the function of VD was partially restored. Similar results were observed in the protein level of fibronectin (Fig. 1c). The fibronectin protein level was increased by HG, which was repressed by VD treatment. When VDR was knocked down by siRNA, the fibronectin protein level was similar as the cells treated by HG only.

Since HG-induced ROS can activate JAK/STAT signaling, which further upregulate fibronectin expression [28, 29], the levels of JAK2 and p-JAK2 were also examined by immunoblotting. As shown in Fig. 1c, increased p-JAK2 level was observed in cells treated by HG. VD treatment repressed p-JAK2 level significantly, which was restored by VDR siRNA. These results indicated that HG upregulated fibronectin expression through increasing ROS level and activating JAK/STAT signaling, which can be repressed by VD treatment.

**Fig. 2:**
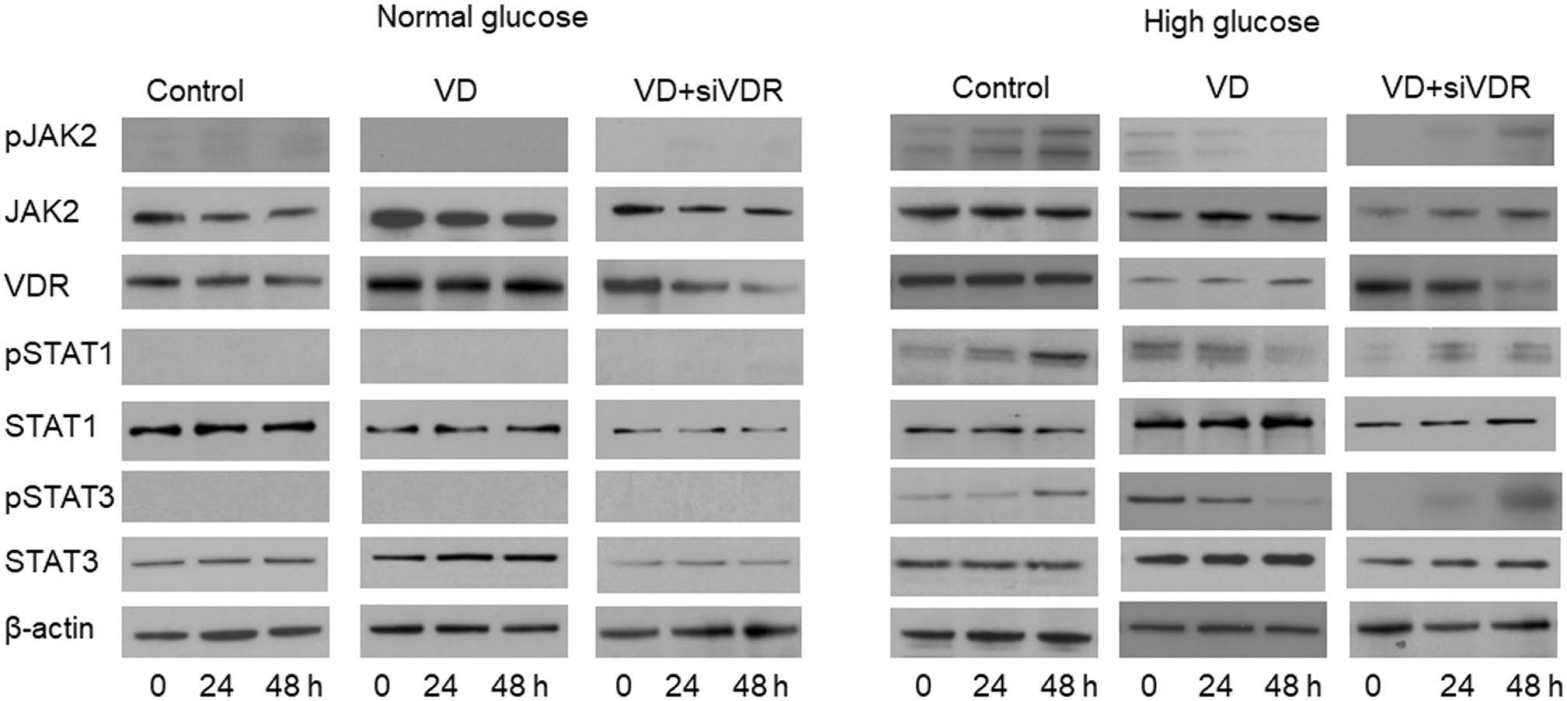
VD inhibited tyrosine phosphorylation of JAK2, STAT1 and STAT3 which was induced by HG. Rat glomerular mesangial cells were cultured under different conditions with or without siVDR transfection for 24 or 48 h, and then subjected to immunoblotting

Subsequently, the level of pJAK2, pSTAT1 and pSTAT3 in glomerular mesangial cells was examined by immunoblotting to confirm the repressive role of vitamin D. As shown in Fig. 2, under NG conditions, there is no tyrosine phosphorylation of JAK2 detected. When glomerular mesangial cells were cultured in HG conditions, p-JAK2 was significantly increased when compared with NG conditions, at both 24 and 48 h, indicating that HG promotes a constitutive activation of JAK2. Meanwhile, VD treatment partially inhibited the HG-induced JAK2 tyrosine phosphorylation at 24 and 48 h. When VDR was knocked down, the inhibition of VD on JAKs phosphorylation was restored.

Similar results were observed in the p-STAT1 and p-STAT3 levels. As shown in Fig. 2, HG induced a significant increased level of phosphorylated STAT1 and STAT3 compared with NG conditions at both 24 and 48 h. Meanwhile, VD repressed the phosphorylated STAT1 and STAT3 level, which was blocked by siVDR. These results indicating that the HG promotes a constitutive activation of JAK/STAT signaling in the absence of growth factors, which can be inhibited by VD.

**Fig. 3:**
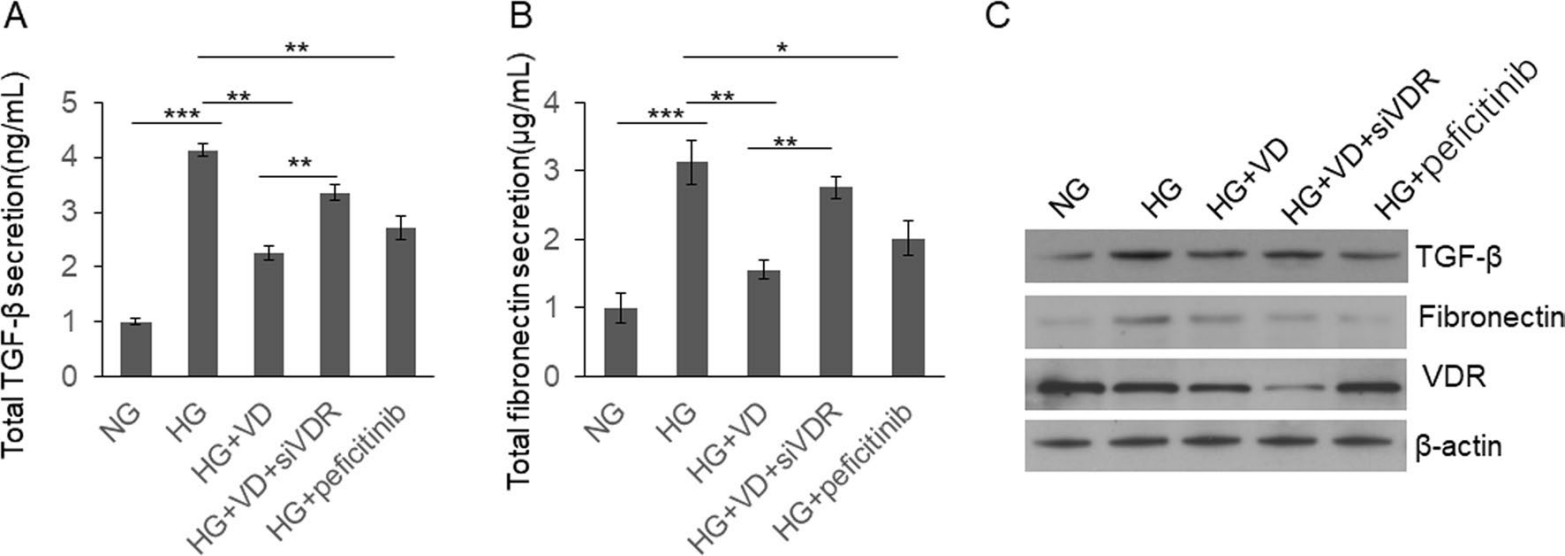
VD inhibited TGF-β and fibronectin secretion by repressing JAK/STAT signaling. Rat glomerular mesangial cells were cultured under different conditions with or without siVDR transfection or peficitinib treatment for 48 h. The cell culture mediums were subjected to ELISA assay to determine secreted TGF-β and fibronectin level. The cells were subjected to immunoblotting. Results were analyzed by one-way ANNOVA and *p* < 0.05 was considered significant. ***p < 0.001, **p < 0.0, *p < 0.05

High glucose was found to induce TGF-β expression, leading to production of extracellular matrix proteins [30]. In systemic sclerosis, VDR was found regulates TGF-β Signaling [31]. To examine whether VD regulate TGF-β Signaling in kidney, we detected the secreted TGF-β and fibronectin levels in the medium by ELISA. As shown in Fig. 3a, b, TGF-β and fibronection levels in the medium were increased significantly in the HG conditions and VD treatment partially repressed production of TGF-β and fibronectin. Meanwhile, siRNA targeting VDR and STAT inhibitor-peficitinib can both partially restored the function of VD. Similar results were observed in the cells. As shown in Fig. 3c, HG treatment upregulated intracellular TGF-β and fibronectin, which was repressed by VD. Si-VDR and peficitinib can both partially restored the function of VD. These results indicated that HG-induced JAK/STAT activation plays an important role in the HG-induced TGF-β synthesis and fibronectin production.

**Fig. 4:**
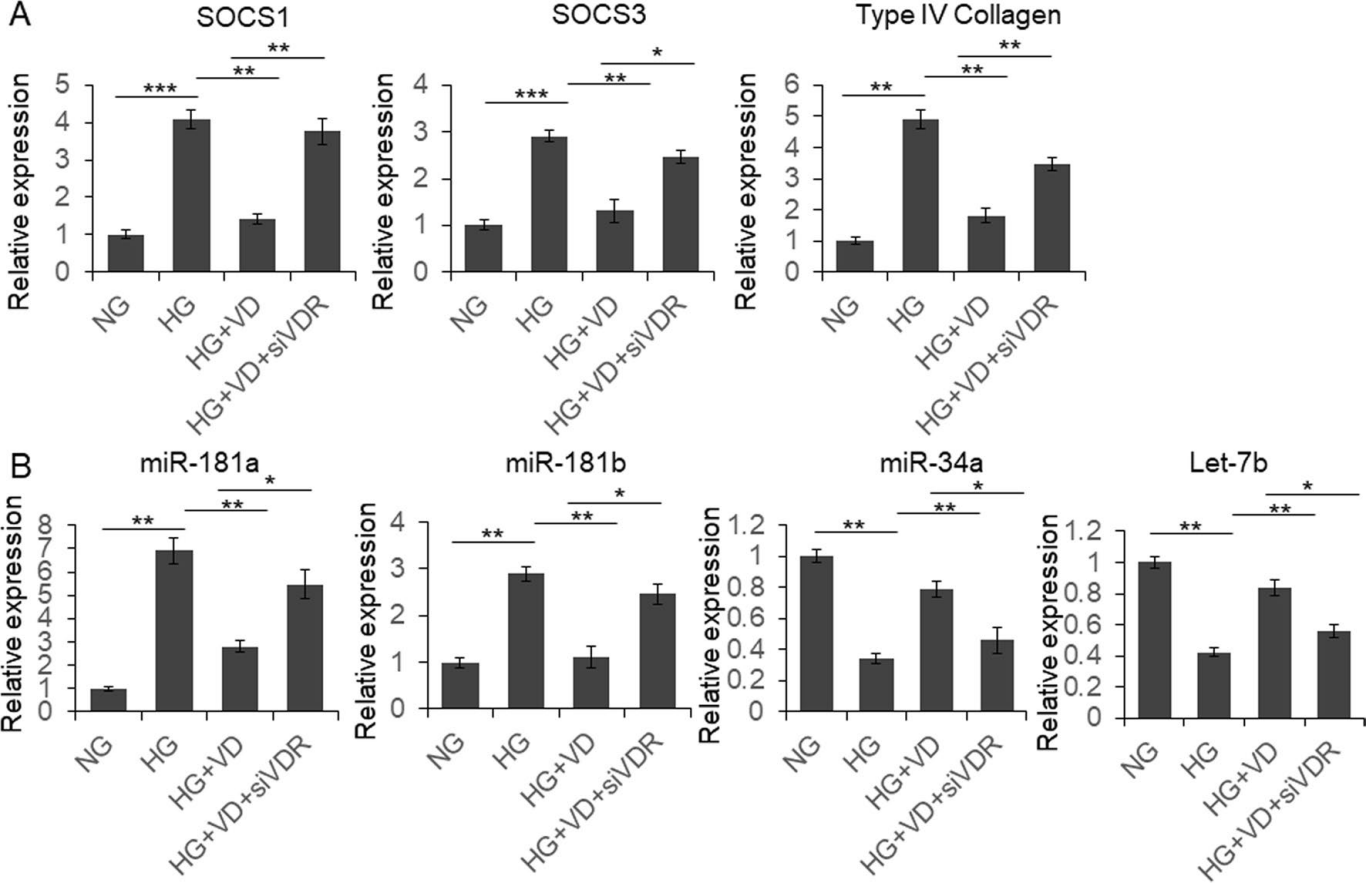
VD regulated the expression of JAK/STAT downstream protein coding genes and miRNAs. Rat glomerular mesangial cells were cultured under different conditions with or without siVDR transfection for 48 h, and then subjected to qRT-PCR. Results were analyzed by one-way ANNOVA and *p* < 0.05 was considered significant. ***p < 0.001, **p < 0.0, *p < 0.05

To further identify the effect of HG on JAK/STAT activation, we examined the expression of JAK/STAT signaling pathway downstream genes, including SOCS1, SOCS3 and type IV collagen. As shown in Fig. 4a, the expression of these three genes were up-regulated under HG condition, and repressed by VD. Meanwhile, many miRNAs were reported dysregulated in patients with DN, but the reason was not well understood [32]. JAK/STAT signaling also participates miRNAs expression regulation. So we detected four reported JAK/STAT downstream miRNAs expression in glomerular mesangial cells cultured under different conditions. As shown in Fig. 4b, the level of miR-181a and miR-181b was increased under HG condition, which was partially restored by VD. The expression of miR-34a and Let-7b was repressed under HG condition, which partially restored by VD.

## Discussion

It is identified that ROS is a common denominator for the causation of renal injury in patients with diabetes. So, ROS has the potential to be a novel target to ameliorate reno-vascular complications of diabetes. ROS can stimulate the activity of STAT kinases and the activation of the JAK/STAT signaling cascade further stimulates excessive proliferation and growth of glomerular mesangial cells, and the matrix proteins expression, contributing to diabetic nephropathy [6, 7]. In the present study, we identified that VD treatment can reduce the amount of HG-induced ROS for the first time, which provides a candidate method for DN treatment.

MiR-181a and miR-181b were reported upregulated by JAK/STAT pathway in breast cancer cells. miR-34a and Let-7b were reported repressed by STAT3 signaling in colon cancer and breast cancer cells [33–36]. However, the relationship between these miRNAs and JAK/STAT signaling is still unknown in glomerular mesangial cells. In this study, we confirmed that HG-induced JAK/STAT activation upregulated the level of miR-181a, miR-181b and repressed the expression of miR-34a and Let-7b, which construct the link between JAK/STAT signaling and these miRNAs in glomerular mesangial cells for the first time.

VD has calciotropic and pleiotropic effects mediated through VDR expressed in kidneys, including podocytes, intestine, bones, parathyroid glands, pancreatic beta cells, monocytes, and T-cells [37]. It is reported that VD can inhibit JAK/STAT signaling in hepatocytes and T cells [23, 24]. However, the relationship between VD and JAK/STAT signaling during DN is still unknown. In this study, we confirmed that VD inhibited HG-induced JAK/STAT signaling activation which provided a candidate choice for clinical DN treatment.

There are limits of this study. (1) This study is just an in vitro study using rat glomerular mesangial cells. These findings need to be confirmed in vivo using diabetic animal models; (2) knockdown VDR just partially restored the function of VD on fibronectin expression, so the full mechanism of VD protective role needs to be unveiled; (3) VD supplementation may be a good choice for diabetic nephropathy prevention, but VD overdose also has side effects. So the use of VD in clinical treatment needs to be studied in depth.

## Supplementary Information

Below is the link to the electronic supplementary material.

Supplementary file1 Fig.S1. VD repressed the level of HG-induced intracellular hydrogen peroxide and superoxide. Rat glomerular mesangial cells were cultured under different conditions with or without siVDR transfection for 48 hours, and then subjected to intracellular hydrogen peroxide (A) and superoxide (B) detection. Results were analyzed by one-way ANNOVA and *p*<0.05 was considered significant. ***p<0.001, **p<0.0, *p<0.05 (TIF 3609 KB)
