# Supplementary figures and images for: Vitamin D protects glomerular mesangial cells from high glucose-induced injury by repressing JAK/STAT signaling
Source: Int Urol Nephrol. 2021 May 3;53(6):1247–54. doi: 10.1007/s11255-020-02728-z (PMC8144147; doi:10.1007/s11255-020-02728-z)

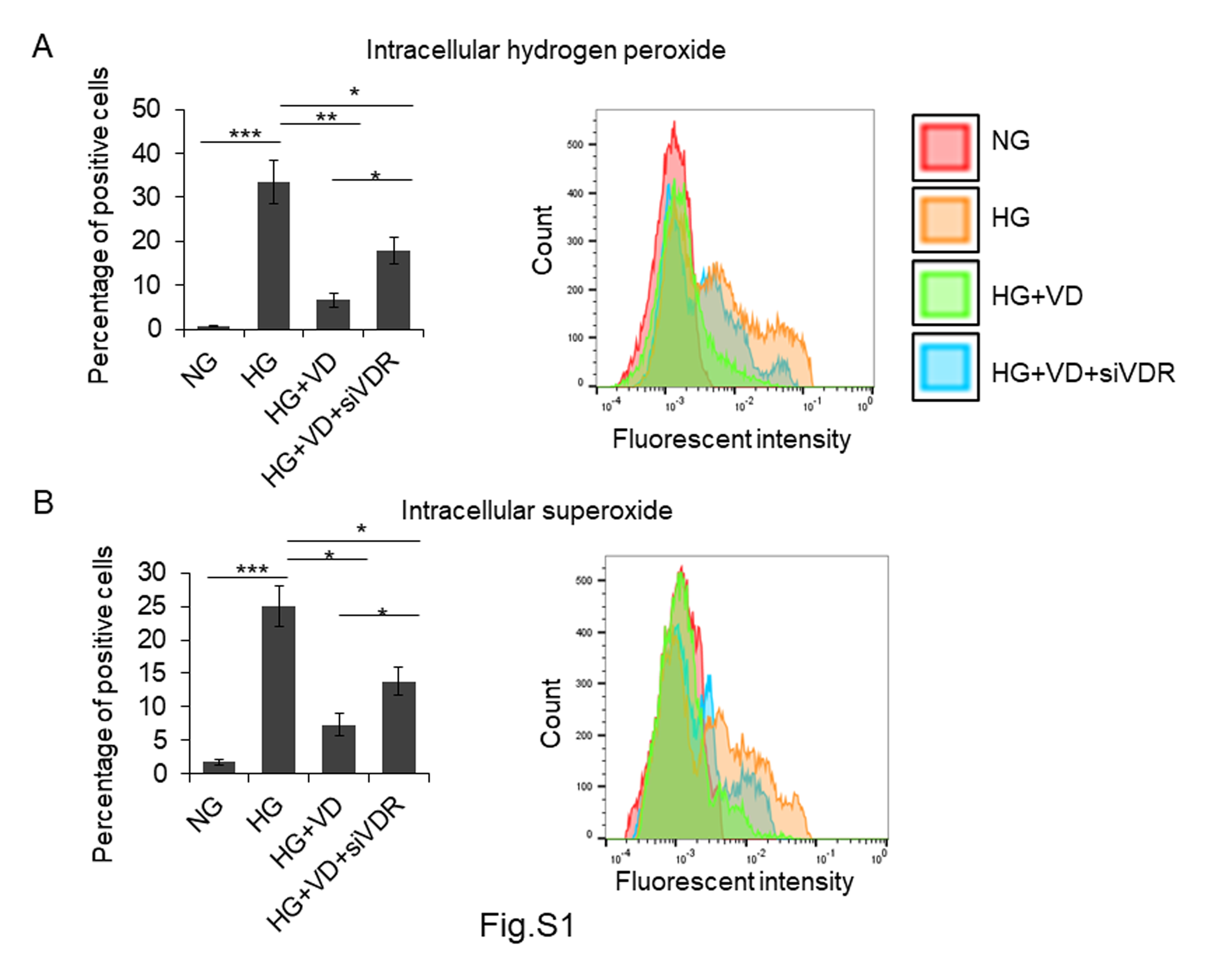

Supplement: Supplementary file 1 — Supplementary file1 Fig.S1. VD repressed the level of HG-induced intracellular hydrogen peroxide and superoxide. Rat glomerular mesangial cells were cultured under different conditions with or without siVDR transfection for 48 hours, and then subjected to intracellular hydrogen peroxide (A) and superoxide (B) detection. Results were analyzed by one-way ANNOVA and p<0.05 was considered significant. ***p<0.001, **p<0.0, *p<0.05 (TIF 3609 KB) [file 11255_2020_2728_MOESM1_ESM.tif]
